# Evaluation of generalization ability for deep learning‐based auto‐segmentation accuracy in limited field of view CBCT of male pelvic region

**DOI:** 10.1002/acm2.13912

**Published:** 2023-01-19

**Authors:** Hideaki Hirashima, Mitsuhiro Nakamura, Keiho Imanishi, Megumi Nakao, Takashi Mizowaki

**Affiliations:** ^1^ Department of Radiation Oncology and Image‐Applied Therapy Graduate School of Medicine Kyoto University Sakyo‐ku Kyoto Japan; ^2^ Department of Advanced Medical Physics Graduate School of Medicine Kyoto University Sakyo‐ku Kyoto Japan; ^3^ e‐Growth Co., Ltd. Kyoto‐shi Kyoto Japan; ^4^ Department of Advanced Medical Engineering and Intelligence Graduate School of Medicine Kyoto University Sakyo‐ku Kyoto Japan

**Keywords:** auto‐segmentation, intensity distribution, limited FOV CBCT, U^2^‐Net CNN

## Abstract

**Purpose:**

The aim of this study was to evaluate generalization ability of segmentation accuracy for limited FOV CBCT in the male pelvic region using a full‐image CNN. Auto‐segmentation accuracy was evaluated using various datasets with different intensity distributions and FOV sizes.

**Methods:**

A total of 171 CBCT datasets from patients with prostate cancer were enrolled. There were 151, 10, and 10 CBCT datasets acquired from Vero4DRT, TrueBeam STx, and Clinac‐iX, respectively. The FOV for Vero4DRT, TrueBeam STx, and Clinac‐iX was 20, 26, and 25 cm, respectively. The ROIs, including the bladder, prostate, rectum, and seminal vesicles, were manually delineated. The U^2^‐Net CNN network architecture was used to train the segmentation model. A total of 131 limited FOV CBCT datasets from Vero4DRT were used for training (104 datasets) and validation (27 datasets); thereafter the rest were for testing. The training routine was set to save the best weight values when the DSC in the validation set was maximized. Segmentation accuracy was qualitatively and quantitatively evaluated between the ground truth and predicted ROIs in the different testing datasets.

**Results:**

The mean scores ± standard deviation of visual evaluation for bladder, prostate, rectum, and seminal vesicle in all treatment machines were 1.0 ± 0.7, 1.5 ± 0.6, 1.4 ± 0.6, and 2.1 ± 0.8 points, respectively. The median DSC values for all imaging devices were ≥0.94 for the bladder, 0.84–0.87 for the prostate and rectum, and 0.48–0.69 for the seminal vesicles. Although the DSC values for the bladder and seminal vesicles were significantly different among the three imaging devices, the DSC value of the bladder changed by less than 1% point. The median MSD values for all imaging devices were ≤1.2 mm for the bladder and 1.4–2.2 mm for the prostate, rectum, and seminal vesicles. The MSD values for the seminal vesicles were significantly different between the three imaging devices.

**Conclusion:**

The proposed method is effective for testing datasets with different intensity distributions and FOV from training datasets.

## 1 INTRODUCTION

In image‐guided radiotherapy, cone beam computed tomography (CBCT) is one of the most widely adopted modalities.^1^, ^2^, ^3^ CBCT can be used not only for patient setup but also for dose evaluation throughout the treatment course. To evaluate daily dose calculations with CBCT, target and organ‐at‐risk segmentation are essential. In general, CBCT images include low soft tissue contrast and image artifacts caused by x‐ray scattering or organ movement during scanning^4^, ^5^; therefore, CBCT segmentation is time‐consuming and its results are highly variable due to inter‐observer error.^6^ Auto‐segmentation is one possible approach to solving these problems.^7^, ^8^, ^9^

Multiple investigators have reported that atlas‐based and convolutional neural network (CNN)‐based segmentation was successfully applied to CT and CBCT images, showing that the CNN‐based methods can provide greater segmentation accuracy and better efficiency than atlas‐based methods.^10^, ^11^, ^12^ Researchers have conducted segmentation of soft tissue organs on CT and/or CBCT using neural encoding/decoding based on the CNN architecture, thereby exploiting supervised training.^13^, ^14^, ^15^, ^16^, ^17^, ^18^, ^19^, ^20^, ^21^ The main requirement for this type of approach is anatomical correspondence between the input image (CT or CBCT), and the ground truth reference label.^13^, ^14^, ^15^, ^16^, ^17^, ^18^, ^19^, ^20^, ^21^ Although CNN‐based segmentation works well for full field of view (FOV) CBCT,^13^, ^14^, ^15^, ^16^, ^17^, ^20^, ^21^ it is unknown whether CNN‐based segmentation using limited FOV CBCT is possible.

Generally, CNNs for medical images perform poorly for new medical images with intensity distributions that are completely different from those of the training dataset.^22^ Furthermore, the trade‐off between FOV size and resolution of the input image remains problematic for segmentation using CNN.^23^ To solve these problems practically, the generalization of new datasets needs to be considered. However, generalization performance for segmentation accuracy would be reduced for different intensity distributions and FOV sizes between the training and testing datasets. As a result, the assessment of generalization performance is crucial when building a model for deep learning.

The aim of this study was to evaluate generalization ability of segmentation accuracy of limited FOV CBCT in the male pelvic region using a full‐image deep neural network. The generalization ability of auto‐segmentation accuracy was evaluated using various testing datasets with different intensity distributions and FOV sizes.

## 2 METHODS

### 2.1 Patient data

A total of 171 patients with prostate cancer who underwent volumetric‐modulated arc therapy were enrolled in this study. Patients who underwent femoral head replacement were excluded. Patients underwent daily CBCT in the supine position. The number of fractions ranged from 35 to 39. One CBCT dataset was extracted from each patient; thus, 171 CBCT datasets were used. A total of 151 CBCT datasets were acquired from Vero4DRT (Hitachi, Ltd., Tokyo, Japan), 10 from TrueBeam STx (Varian Medical Systems, Palo Alto, CA, USA), and 10 from Clinac‐iX (Varian Medical Systems). Detailed specifications for the three CBCT devices and exposure parameters are presented in Table 1. The FOVs for Vero4DRT, TrueBeam STx, and Clinac‐iX were 20, 26, and 25 cm in diameter, respectively. The regions of interest (ROIs) of the bladder, prostate, rectum, and seminal vesicles were manually delineated by an experienced medical physicist to reduce inter‐observer error. An experienced medical physicist received a prior lecture from radiation oncologists regarding contouring. This study was approved by our institutional review board (R1499) and adhered to all relevant ethical tenets of the Declaration of Helsinki.

**TABLE 1:** Detailed specifications for the CBCT imaging devices

Device	Field of view [cm]	Matrix size	Resolution [mm] (X × Y × Z)	Kilovoltage peak (kVp)	Current (mA)
Vero4DRT	***φ*** 20	512	0.39 × 0.39 × 2.0	110–120	160–200
TrueBeam STx	***φ*** 26	512	0.51 × 0.51 × 2.0	125	80
Clinac‐iX	***φ*** 25	384	0.65 × 0.65 × 2.5	125	80

### 2.2 Network architecture

**FIGURE 1:**
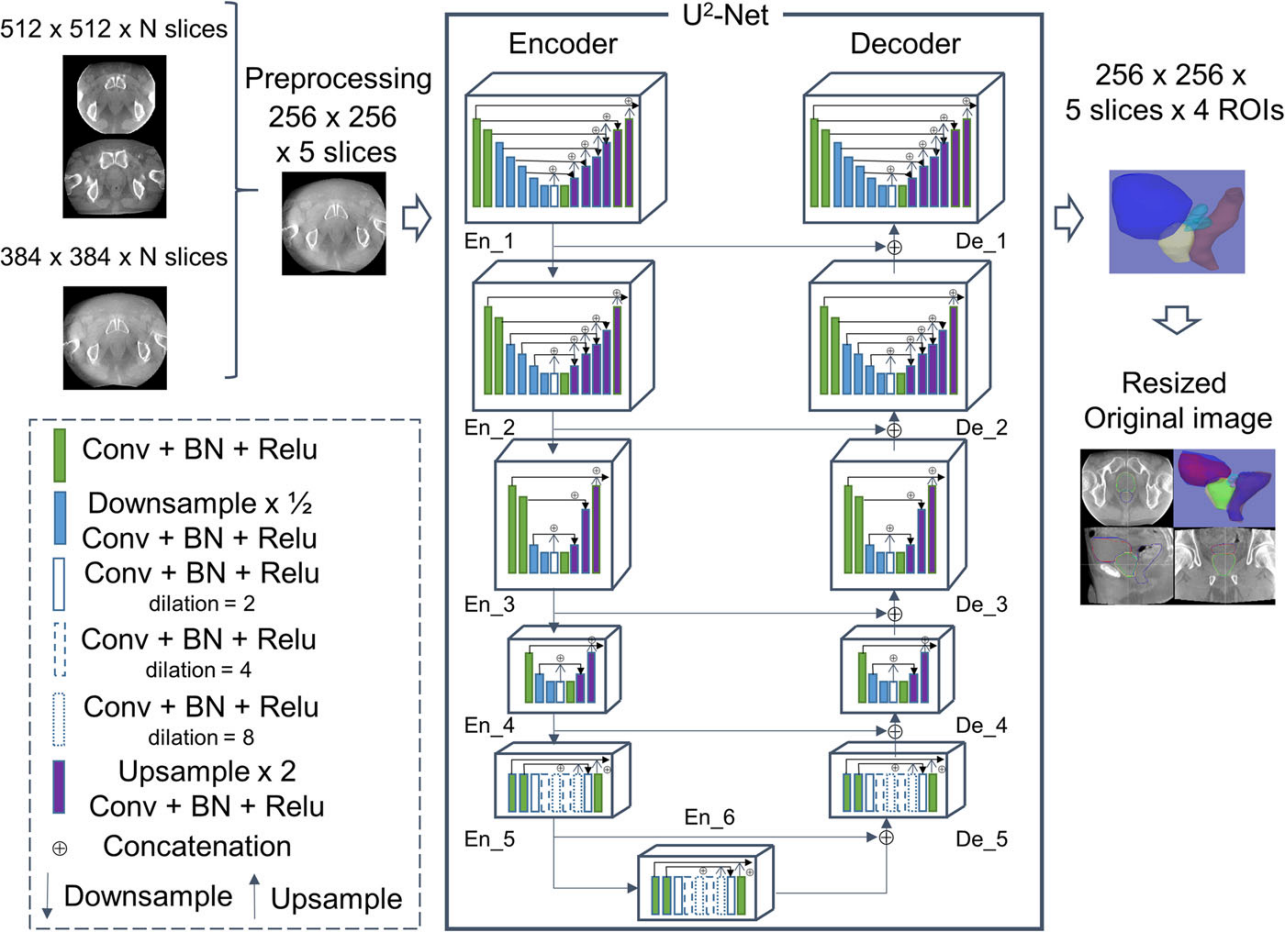
U^2^‐Net architecture schematic. The CNN architecture U^2^‐Net was used, which was constructed by using a double‐nested U‐Net. The main architecture is a U‐Net‐like Encoder‐Decoder, where each stage consists of our newly proposed residual U‐block.^24^ Conv, convolution filter; BN, batch normalization; Dilation, dilation of convolution filter

The CNN architecture U^2^‐Net was used, which was constructed by using a double‐nested U‐Net (Figure 1).^24^ Its top level is a big U‐structure that consists of 11 stages. Each stage is filled by a well configured residual U‐block. Both local and global contextual information are important for salient object detection and other segmentation tasks. Thus, network architecture comprising a residual U‐block was proposed, and the details of the nested U‐architecture built with this block featured more efficient local and global information extraction. The design of U^2^‐Net allowed for a deep architecture with rich multiscale features and relatively low computational and memory costs. The U^2^‐Net architecture was built using only residual U‐blocks without any pretrained backbones adapted from image classification and was easily adaptable to different working environments without significant performance loss. After applying the convolution layer and sigmoid activation to the saliency maps at each stage (En_6, De_5, De_4, De_3, De_2, and De_1) of U^2^‐Net, the output at each deconvolution layer becomes the output enlarged to the size of the input image. The resolution of the feature maps in En_6 is relatively low, and further down‐sampling of these feature maps leads to a loss of useful context.^24^ Thus, a saliency map with additional useful information was constructed by combining feature maps at states where the resolution is up‐sampled (De_5, De_4, De_3, De_2, and De_1). These are combined, and finally the convolution layer and sigmoid function upsample these saliency maps to the input size and fuse them with a concatenation operation to generate the input image size. Hence, the nested U‐structure enabled a more efficient extraction of intra‐ and inter‐stage local and global features.

To obtain better segmentation accuracy, all input images were reconstructed from their original matrix size to a 256 × 256 matrix size, and volume‐to‐volume translation was performed in the model. Hence, the input image matrix size was 256 × 256 for five slices. The output image matrix size was 256 × 256; five slices and four labels (contours). Thereafter, output images were resized to the original image.

### 2.3 Model building

**FIGURE 2:**
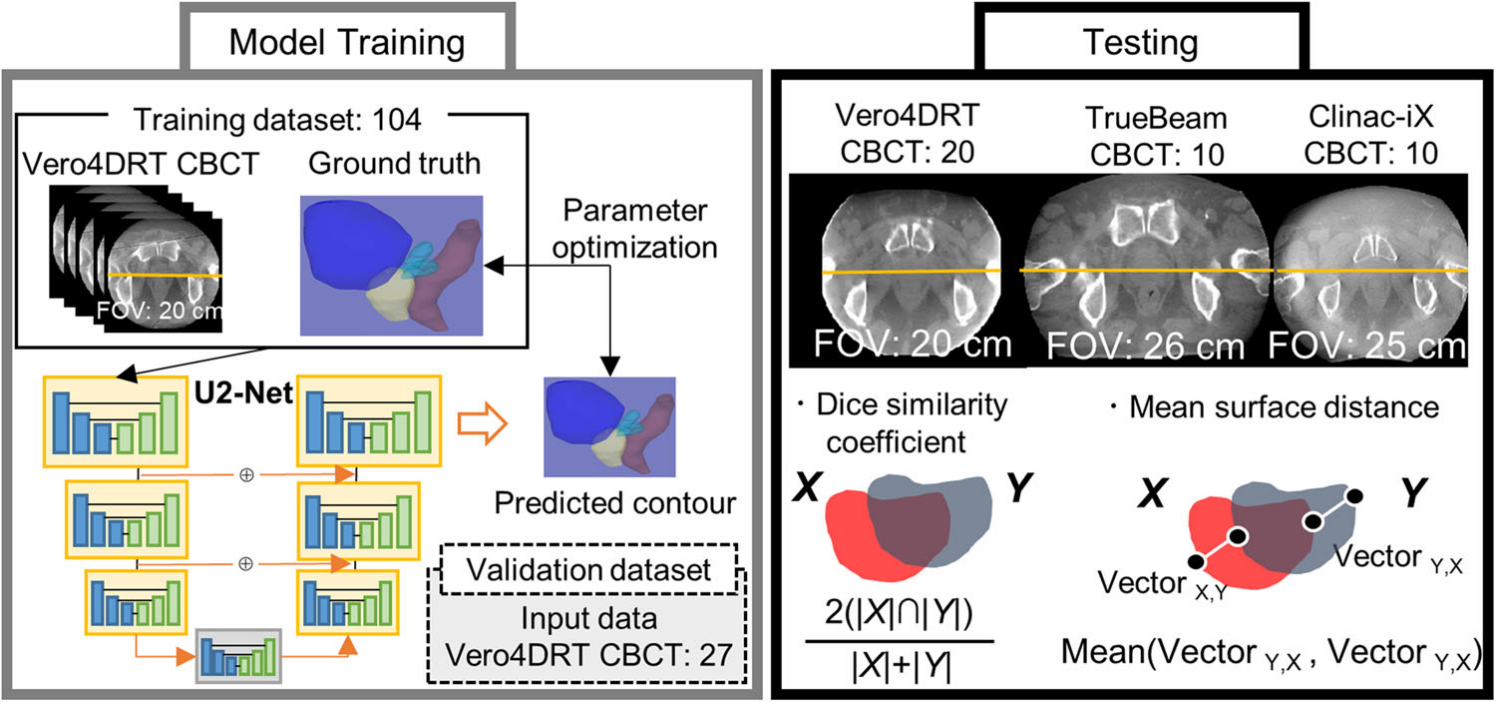
Overall study flow. The left side shows the training phase, and the right side shows the testing phase. The U^2^‐Net architecture in the training phase was drawn in a simplified manner

Figure 2 illustrates the overall flow used in this study. Datasets were separated as follows: training dataset, 104 limited FOV CBCTs (Vero4DRT); validation dataset, 27 limited FOV CBCTs (Vero4DRT); and testing dataset, 40 limited FOV CBCTs (20 from Vero4DRT, 10 from TrueBeam STx, and 10 from Clinac‐iX). First, real‐time image augmentation was performed during training using Growth RTV software (e‐Growth Co., Ltd., Hyogo, Japan), feeding the network with random three‐dimensional (3D) rotations within ± 15° and random multi‐window settings with window levels of –100 to 100 Hounsfield unit (HU) and window widths of 1500 to 2000 HU. Second, corresponding patches from the limited FOV CBCT and ground truth segmentation in five consecutive axial planes were randomly extracted. The loss function calculated the dice similarity coefficient (DSC) between the ground truth and predicted segmentation. Optimization metrics were calculated using Adam with a learning rate of 5.0 × 10^−5^. The exponential decay rate for the first‐moment estimate and the second‐moment estimate was 0.9 and 0.999, respectively. The exponential decay rates for the first‐moment and the second‐moment estimates were defined that the hyperparameter which determines how much to change is defined as the decay rate. The value of the decay rate was reported in a previous paper.^24^ Weight decay was set to 0. The training routine was set to save the best weight values when the DSC in the validation set was maximized.

The network model, loss function, and evaluation metrics were built using Keras (version 2.4.3) and TensorFlow (version 2.4.0) frameworks in Python (version 3.7). The training was conducted in a workstation equipped with a 6‐core CPU, 64 GB RAM, and NVIDIA RTX 8000 with 48 GB RAM. The network was trained for 1000 epochs. Batch size was eight for less than 100 epochs, 10 for l00–200 epochs, 12 for 200–300 epochs, 14 for 300–400 epochs, 16 for 400–500 epochs, 18 for 500–600 epochs, and 20 for 600–1000 epochs. Training time required approximately 40 h. The testing was conducted using the Growth RTV software training model, in a workstation equipped with an 8‐core CPU, 32 GB RAM, and 11 GB NVIDIA GeForce RTX 2080 Ti support.

### 2.4 Evaluation

To determine the quality of auto‐segmentation, a visual evaluation was performed. According to the AAPM TG‐132, a score between 0 and 4 points is assigned to the segmentation accuracy; 0 point, no need for modification contour; 1 point, modification contour by 25%; 2 points, modification contour by 50%; 3 points, modification contour by 75%; 4 points, the necessity of modification whole contour.^25^ A visual evaluation was performed in each organ. We computed the mean values and standard deviation (SD) of the visual evaluations for all organs. Further, segmentation accuracy was evaluated with 3D DSC and 3D mean surface distance (MSD) between the ground truth and predicted ROIs for the different testing datasets, with 3D MSD representing the mean value of the nearest bidirectional point‐to‐surface distance.^26^ Statistical analysis was performed using EZR (Saitama Medical Center, Jichi Medical University, Saitama, Japan) to calculate the analysis of variance and estimate the DSC and MSD.^27^ The level of significance was set at *p* < 0.05.

## 3 RESULTS

**FIGURE 3:**
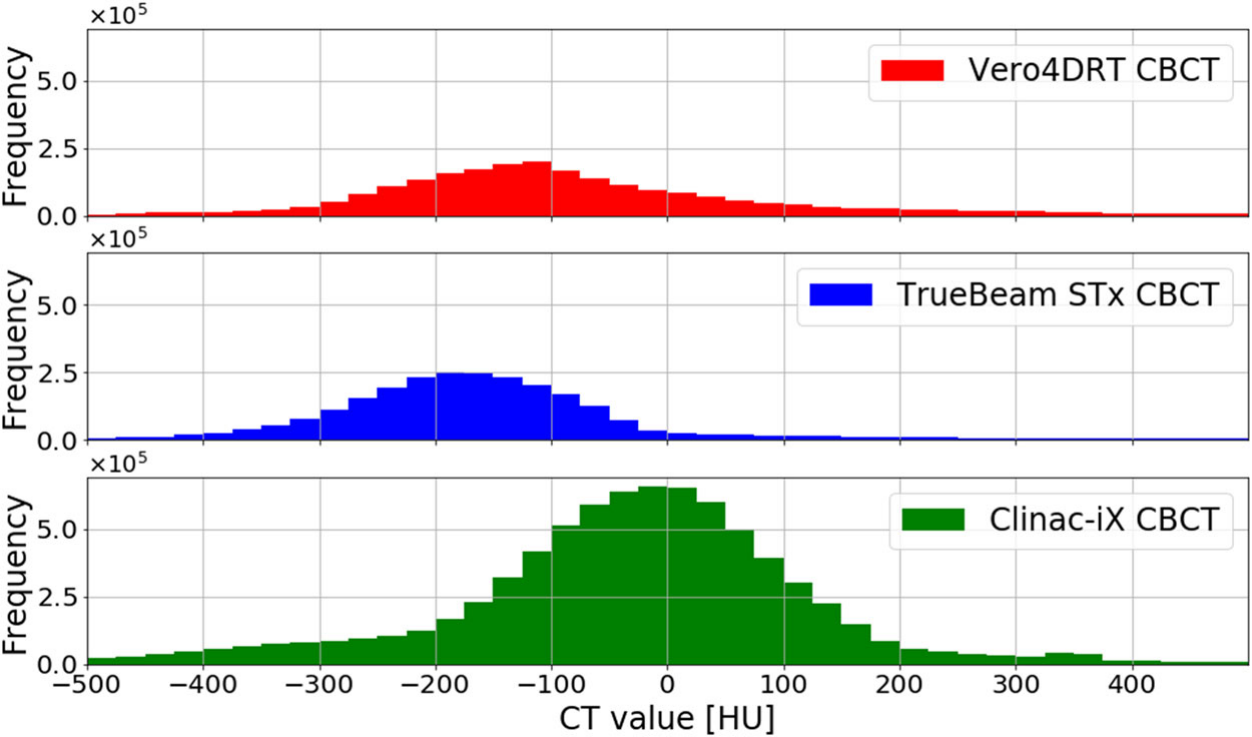
A histogram of intensity distributions for Vero4DRT CBCT, TrueBeam STx CBCT, and Clinac‐iX CBCT in three representative patients. The histogram's CT values are shown ranging from –500 to 500 Hounsfield units. CBCT, cone beam computed tomography

The time required for predicting four ROIs was 30 s per patient. The mean scores ± SD of visual evaluation for bladder, prostate, rectum, and seminal vesicle in all treatment machine were 1.0 ± 0.7, 1.5 ± 0.6, 1.4 ± 0.6, and 2.1 ± 0.8 points, respectively. A histogram comparing image intensity distributions for the three CBCT devices in the representative patients is presented in Figure 3. The histogram peak for Vero4DRT, TrueBeam STx, and Clinac‐iX CBCT was observed at –175, –100, and 0 HUs, respectively.

**FIGURE 4:**
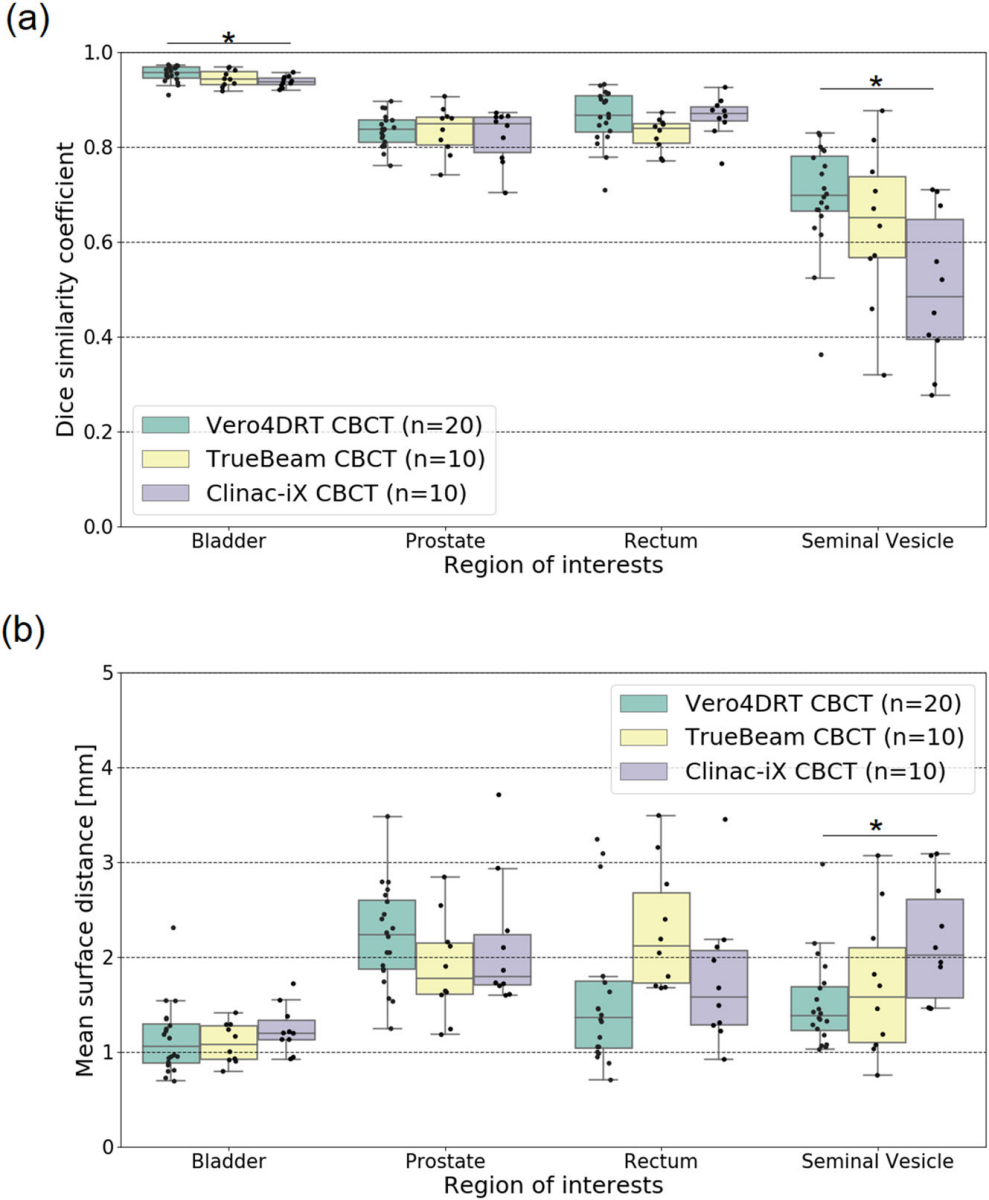
Boxplot of dice similarity coefficients and mean surface distances for the bladder, prostate, rectum, and seminal vesicles. The asterisk shows a statistical significant difference by analysis of variance method

**FIGURE 5:**
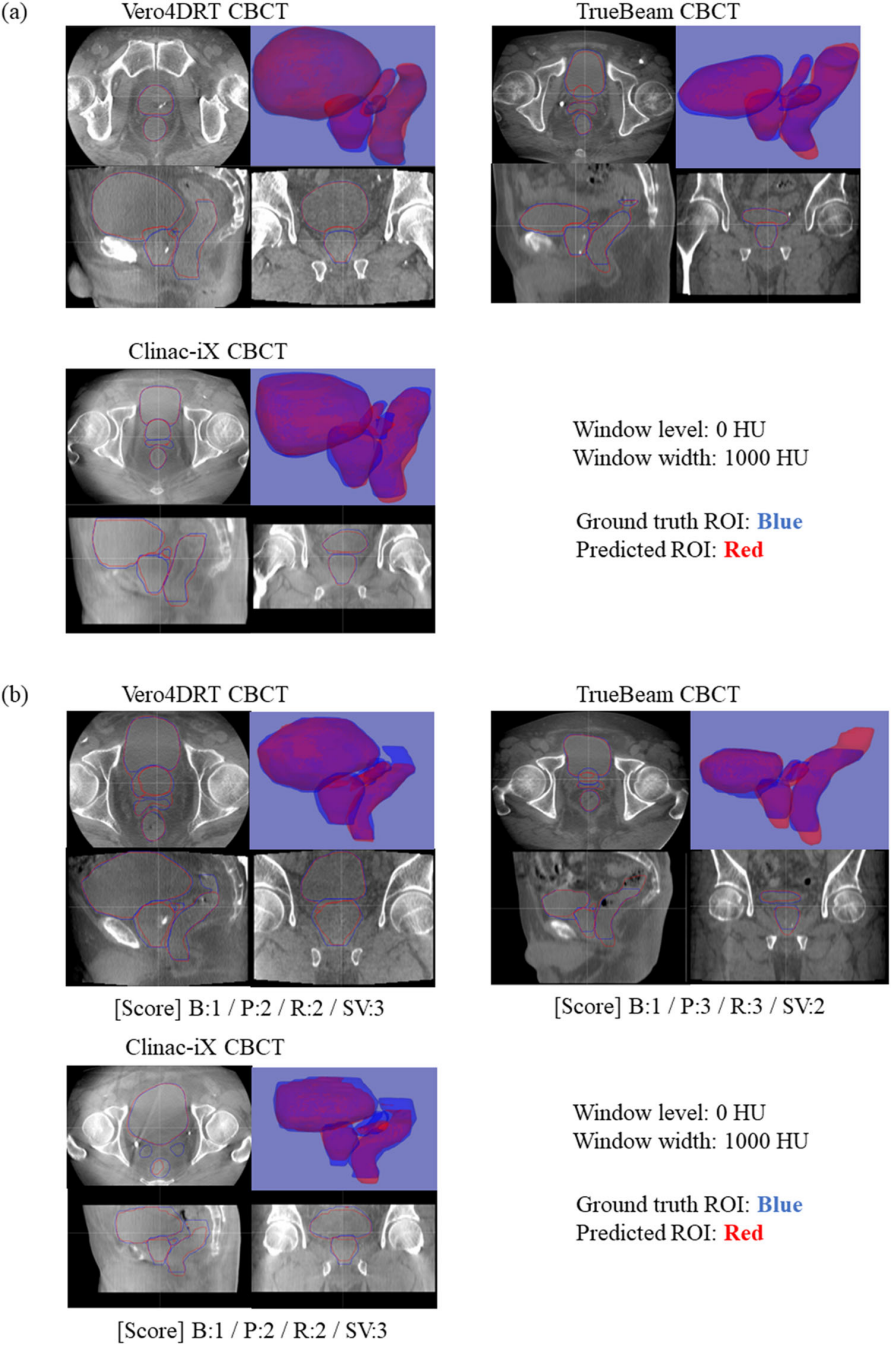
The representative visual results for the ground truth (blue) and predicted ROIs (red). (a) Representative segmentation results and (b) the worse segmentation results in Vero4DRT, TrueBeam STx, and Clinac‐iX CBCT systems. The scores of visual evaluation for bladder, prostate, rectum, and seminal vesicle are shown below the figure. CBCT, cone beam computed tomography; B, bladder; P, prostate; R, rectum; SV, seminal vesicle

The histogram shapes of all imaging systems were found to be unimodal, but the peak positions were different. The segmentation accuracy of the testing dataset is summarized in Figure 4. The median DSC values for all imaging devices were ≥ 0.94 for the bladder, 0.84–0.87 for the prostate and rectum, and 0.48–0.69 for the seminal vesicles. Although the DSC values for the bladder and seminal vesicles were significantly different among the three imaging devices (*p* < 0.05), the DSC value of the bladder changed by <1% point. The median MSD values for all imaging devices were ≤1.2 mm for the bladder and 1.4–2.2 mm for the prostate, rectum, and seminal vesicles. The MSD values for the seminal vesicles were significantly different between the three imaging devices (*p* < 0.05). Figure 4 shows that the DSC and MSD values for the Vero4DRT dataset were consistently comparable or superior to those for the TrueBeam STx and Clinac iX datasets. The largest variations were observed for the seminal vesicles using Clinac‐iX, where the median DSC and MSD values were 0.48 and 2.0 mm, respectively. Overall, the model showed consistent performance among the various datasets. Figure 5 shows the representative visual results for the ground truth and predicted segmentations. All ROIs with the worse segmentation accuracy had pronounced differences in the superior and inferior directions (Figure 5b).

## 4 DISCUSSION

In this study, we developed a U^2^‐Net‐based automatic segmentation CNN model for limited FOV CBCT and used it to evaluate male pelvic organs, including the bladder, prostate, rectum, and seminal vesicles. Table 2 summarizes the comparison of deep learning‐based auto‐segmentation results reported by several previous studies and the present study. Several studies obtained mean DSC values of 0.84–0.96 for the bladder, 0.73–0.91 for the prostate, 0.70–0.93 for the rectum, and 0.70 for the seminal vesicles using full FOV CBCT. These results were comparable to our findings.^13^, ^14^, ^15^, ^16^, ^17^, ^19^, ^20^, ^21^

**TABLE 2:** Summary comparing pelvic region segmentation accuracy between previous studies and this study. Mean values for dice similarity coefficients and mean surface distances [mm] are shown

								Regions of interest			
Author	Image	Model	Patients	Training	Validation	Testing	Evaluation Method	Bladder	Prostate	Rectum	Seminal vesicle
Fu^13^	Full FOV CBCT Synthetic MRI	CycleGAN	100	Five‐fold CV			DSC (2D)	0.96	0.91	0.93	
							MSD (2D)	0.65	0.93	0.72	
Brion^14^	Full FOV CBCT	3D U‐Net	134	74		60	DSC (3D)	0.81	0.73	0.65	
							MSD (3D)	4.00	3.63	5.78	
Zhou^15^	CT Full FOV CBCT	2.5D YOLO	85	65	10	10	DSC (3D)	0.92		0.78	
							MSD (3D)	1.68		2.90	
Leger^16^	CT Full FOV CBCT	3D U‐Net	63	Three‐fold CV			DSC (2D)	0.87	0.76	0.81	
							MSD (2D)	2.47	3.08	2.38	
Liu^17^	Full FOV CBCT	Multi‐class Segmentation U‐Net	105	77	14	14	DSC (2D)	0.92		0.79	
Hirashima^19^	CT	FusionNet	470	270	90	90	DSC (3D)	0.95	0.82	0.89	0.71
Shreier^20^	CT pseudo CBCT	BibNet	350	300 (CT) 300 (pseudo CBCT)		5 (CBCT) 45 (CT)	DSC (3D)	0.93	0.84	0.87	0.70
Brion^21^	Full FOV CBCT	3D FCN	48	Six‐fold CV			DSC (2D)	0.80			
Present study	Limited FOV CBCT	U^2^‐net	171	104 (FOV 20 cm)	27 (FOV 20 cm)	20 (FOV 20 cm)	DSC (3D)	0.95	0.83	0.86	0.69
							MSD (3D)	1.03	2.22	1.73	1.50
						10 (FOV 26 cm)	DSC (3D)	0.94	0.83	0.83	0.64
							MSD (3D)	1.09	1.88	2.29	1.70
						10 (FOV 25 cm)	DSC (3D)	0.93	0.82	0.86	0.48
							MSD (3D)	1.23	2.12	1.75	2.15

Abbreviations: 2D, two dimensional; 3D, three dimensional; CBCT, cone beam computed tomography; CT, computed tomography; CV, cross validation; CycleGAN, cycle generative adversarial network; DSC, Dice similarity coefficient; FCN, fully convolutional neural network; FOV, field of view; MRI, magnetic resonance imaging; MSD, mean surface distance in millimeter; YOLO, you only look once.

Segmentation accuracy would be varied by intensity of image, FOV, and their combination. In this work, we discuss the effect of image intensity on the accuracy of segmentation. Especially, the effect of different intensity distributions on segmentation accuracy could be significantly different in bladder and seminal vesicles (*p* < 0.05); however, no significant difference was appreciated among the other ROIs. Hirashima et al. stated that segmentation using CNN is not optimal for small ROIs.^19^ Nevertheless, the median DSC value in this study was comparable to those reported in previous studies. In general, intensity distributions for medical images are dependent on the characteristics of the imaging device. The performance of models trained on datasets with intensity distributions considerably different from those of the testing datasets is poor.^22^ However, we found that the segmentation performance was comparable among the three imaging devices imaging devices regardless of different intensity distributions (Figure 4). The DSC was improved adjusting the window level and width in the preliminary experiment.^28^ This could indicate that the differences in intensity shown in Figure 3 were canceled out in the training process because of random multi‐window settings during image augmentation.

Although the segmentation accuracy of the bladder and seminal vesicles varied slightly because of the difference in FOV, it is thought to have little effect on the geometric metrics, such as the DSC and MSD. The convolution filter has a process for extracting and determining the features of surrounding pixels. With a normal U‐net, the network becomes more complex and computation time increases greatly; alternatively, the resolution can decrease due to down‐sampling,^24^ resulting in reduced segmentation accuracy. Therefore, we expected that differences in FOV would affect prediction accuracy due to the presence of pixel features that are steeper than the body contour boundaries. However, the contours delineated in this study were located near the center of the image and were not affected by the steep pixel value changes at the image edges. By nesting the networks with additional residual blocks in U^2^‐Net, the model capacity did not become too large, and we believe this factor contributed to efficient learning, improved segmentation accuracy, and overall high performance. Furthermore, in this study the input image resolution was unified to a matrix size of 256 × 256 in preprocessing. Thus, the accuracy of contour delineation may not have been affected by differences in FOV.

Our study has some limitations. First, the accuracy of ground truth segmentation was not perfect. The original CBCT images had low contrast; thus, manual contouring errors still remain. Second, the number of test datasets was small and the number of test datasets was not equivalent between the three CBCT devices. No further data collection from TrueBeam and Clinac‐iX was possible owing to operational difficulties in our department.

## 5 CONCLUSIONS

This study developed a U^2^‐Net‐based automatic segmentation CNN model, and its performance was first evaluated with limited FOV CBCT in the male pelvic region. Our proposed method is effective for testing datasets with different intensity distribution and FOV from training datasets, and provided comparable results to those of previous studies.

## AUTHOR CONTRIBUTIONS

Hideaki Hirashima and Mitsuhiro Nakamura (corresponding author) planned the study, performed the statistical analysis, and drafted the manuscript. Keiho Imanishi, Megumi Nakao, and Takashi Mizowaki conceived the study, participated in its design and coordination, and helped draft the manuscript. All authors read and approved the final manuscript.

## CONFLICT OF INTEREST

The authors have no relevant conflicts of interest to disclose.

## Data Availability

Data available on request from the authors.
